# Redesigning Flexible Food Packaging for the Circular Economy: A Comparative Assessment of Hybrid Paper-Based Solutions

**DOI:** 10.3390/polym18101197

**Published:** 2026-05-13

**Authors:** Johnatan Gabriel Bernal-Carrillo, Mariamne Dehonor-Gómez, Luis E. Lugo, Ana del Carmen Susunaga Notario, Víctor Hugo Mercado-Lemus, José Antonio Betancourt-Cantera, Raúl Pérez-Bustamante, John Edison García-Herrera, Hugo Arcos-Gutiérrez, Isaías E. Garduño

**Affiliations:** 1 Posgrado CIATEQ A.C., Eje 126 No. 225, Zona Industrial del Potosí, San Luis Potosí 78395, Mexico; johnatan.bernal@icloud.com; 2 CIATEQ, A.C., Circuito de la Industria Poniente No. 11 Lote 11 Mz 3, Parque Industrial Ex Hacienda, Lerma de Villada 52004, Mexico; mariamne.dehonor@ciateq.mx (M.D.-G.); luis.lugo@ciateq.mx (L.E.L.); 3 Secihti—ICAT Instituto de Ciencias Aplicadas y Tecnología, Universidad Nacional Autónoma de México, Circuito Exterior S/N, Ciudad Universitaria, Coyoacán, México City 04510, Mexico; ana.susunaga@icat.unam.mx; 4 Secihti—InnovaBienestar de México, Ciencia y Tecnología 790, Saltillo 400, Saltillo 25290, Mexico; victor.mercado@innovabienestar.mx (V.H.M.-L.); jbetancourt@innovabienestar.mx (J.A.B.-C.); 5 Secihti—Centro de Investigación en Materiales Avanzados A.C. (CIMAV), Av. Miguel de Cervantes Saavedra 120, Complejo Industrial Chihuahua, Chihuahua 31136, Mexico; raul.perez@cimav.edu.mx; 6 Secihti—CIATEQ A.C., Eje 126 No. 225, Zona Industrial del Potosí, San Luis Potosí 78395, Mexico; john.garcia@ciateq.mx (J.E.G.-H.); hugo.arcos@ciateq.mx (H.A.-G.)

**Keywords:** flexible packaging, Doypack^®^, hybrid packaging, circular economy, anaerobic degradation, paper-polymer laminate

## Abstract

The increase in environmental impacts associated with non-recyclable flexible plastic packaging underscores the need for sustainable alternatives. This work presents a new paper-based laminate containing cellulose, ethylene-vinyl alcohol (EVOH), and an anaerobic degradation additive (ECO-ONE^®^) to replace existing trilaminate plastics for dry food applications. The hybrid package maintains consistent mechanical performance compared to conventional structures while improving barrier properties: oxygen transmission rate decreased from 35.38 ± 2.1 to 0.56 ± 0.05 cm^3^/m^2^/day (0% RH), and water vapor transmission rate decreased from 4.85 to 1.22 g/m^2^/day. The hybrid structure uses 37% less virgin resin and reduces adhesive/solvent use by 50%. Life cycle assessment indicates a 47% reduction in environmental impact. Approximately 85–90% of waste avoidance is attributable to reduced virgin plastic and adhesive use, with 10–15% attributable to end-of-life treatment. This study presents a practical transitional alternative for dry food packaging applying circular economy principles.

## 1. Introduction

An essential characteristic of modern industrial production is the exponential increase in the use of plastic products over the past few decades [1]. The packaging industry, which is a major driver of plastic consumption, particularly in the food and beverage industry, has experienced a rapid increase in demand for flexible packaging or flexible containers produced from plastics due to their key advantages, including low weight, durability, and high-barrier characteristics necessary for preserving the unique identity and safety of packaged products [2,3,4]. Although flexible packaging has these advantages for product preservation and maintaining product integrity, the linear consumption model utilized in the large majority of cases (characterized by short useful life of the product and a lack of effective recovery mechanisms) has resulted in the creation of substantial environmental burden; it is estimated that approximately 79% of plastic waste is generated and disposed of in landfills or to surrounding natural ecosystems, illustrating the need to generate and implement innovative end-of-life solutions that can be implemented on a large scale [5,6].

Reforming product design and material production to promote reuse and recycling within the framework of a circular economy offers a potential avenue to reducing the current environmental crisis associated with food packaging [7,8,9,10,11]. However, several technical obstacles must be overcome to incorporate sustainable packaging alternatives that meet all relevant packaging performance criteria [12,13]. Any new flexible packaging products produced as an alternative to existing flexible packaging products must demonstrate mechanical performance exceeding established industry standards [14] and meet all thermal and barrier performance requirements.

When developing a new package design, key attributes determine its success in our industry. New packages must meet rigorous testing requirements for mechanical strength [14], thermal acclimation, and barrier protection to ensure shelf stability and provide a safe product [15]. Additionally, the new package designs must be economically viable for mass production [16]. Past research has explored many different avenues, such as material substitution [1,17], down gauging [18], and creating and developing bio-based materials [19]; however, they have typically resulted in some level of compromise due to cost [20], functional performance [21], or developing large-scale manufacturing capabilities [22,23].

Building on the current willingness of consumers to support sustainable package redesigns, which is at an all-time high [1]. This study aims to determine the feasibility of developing a high-performing hybrid paper-based package design. An obstacle when implementing paper-based hybrid packaging as a high-performance barrier package or application, such as a stand-up pouch (commercially recognized as a Doypack^®^) [24], is maintaining the barrier properties of that hybrid construction under mechanical stress. The geometry of the Doypack^®^, or its unique bottom gusset, imposes a specific flexural stress on the packaging during filling and distribution.

Despite their promise, hybrid paper-polymer materials present several limitations that must be acknowledged. The heterogeneous composition complicates end-of-life management: consumers may incorrectly classify such materials as “paper,” leading to plastic contamination of paper recycling streams [24]. Conversely, when directed to plastic recycling, the cellulose fibers can cause processing difficulties and quality reduction in recycled polymers. Furthermore, the partial recyclability achieved in this study (78.5% fiber recovery) still generates 21.5% residual plastic waste that requires separate management. These challenges position hybrid laminates as transitional solutions rather than final circular-economy endpoints.

The novelty of this work lies in three specific aspects: (1) the integration of a continuous LDPE/EVOH co-extruded barrier layer with paper substrate in a Doypack^®^ geometry, where the LDPE (Low-density polyethylene) layer simultaneously protects the moisture-sensitive EVOH core and provides product-contact compliance; (2) the incorporation of ECO-ONE^®^ anaerobic degradation additive specifically designed for landfill conditions, with quantitative 90-day degradation data (34.2%); and (3) the systematic combination of mechanical, barrier, LCA (Life cycle assessment), and end-of-life analyses for a stand-up pouch application, providing industrial-ready validation. While paper-polymer laminates exist commercially, most lack the high oxygen barrier (OTR < 1 cm^3^/m^2^/day) demonstrated here or have not been validated for the specific mechanical demands of Doypack^®^ geometry.

This research aims to fill a gap by developing a material combination that eliminates the need for fossil-based PET (Polyethylene terephthalate) layers, replacing them with a bio-based cellulose substrate. Specifically, the design provides coaction between the fibrous paper and a coextruded LDPE/EVOH matrix at their interface. In addition to this, a 50-micrometer-thick LDPE adhesive sealant has been put in direct contact with the product, allowing it to protect the moisture-sensitive EVOH core, thus creating a hybrid system that achieved an important milestone by providing a reduction in oxygen transmission rate (OTR) from an initial baseline of 35.38 ± 2.1 to 0.56 ± 0.05 cm^3^/m^2^/day at 0% relative humidity.

This paper examines whether such a redesign will improve recycling and degradability without negatively impacting critical functional properties, providing an established pathway towards a 37% reduction in virgin plastic and an overall 47% reduction in environmental impacts.

## 2. Materials and Methods

This research investigates the sustainable redesign of two plastic packaging types: a laminated coil and a stand-up pouch (commercially known as Doypack^®^) [24]. A comparative evaluation was conducted between conventional packaging structures and proposed environmentally conscious alternatives, focusing on key mechanical properties. These included thickness, weight, lamination strength, seal durability, tensile strength, elongation, friction coefficient, oxygen transmission rate (OTR), and water vapor permeability (WVTR). Destructive tests were also conducted to assess mechanical resistance, including impact resistance, atmospheric pressure, and hermeticity. These products are manufactured by converting plastic films through rotogravure printing, lamination (with or without solvents), cutting, and forming. The laminated coil consists of printed and laminated plastic films that are subsequently used to form bags or other flexible packaging. Unlike the coil, the Doypack^®^ is a preformed bag sealed with temperature- and pressure-sensitive seals. A Doypack^®^ is a stand-up pouch made of flexible, multilayered materials designed to protect and store various products. Its unique design includes a bottom gusset that allows the pouch to stand upright on shelves, making it an excellent option for branding and practicality. Doypack^®^ is a trademarked brand name for a packaging design created in 1962 by French inventor Louis Doyen. Although Doypack^®^ is a trademark, the term has become widely used to refer to this type of stand-up pouch made of flexible plastic, even when not referring to the officially trademarked product. It is now a general term in the packaging industry [24].

### 2.1. Selection of Conventional Baseline and Study Framework

The conventional trilaminate structure (PET/ink + adhesive/PET/adhesive/LDPE) was selected as the baseline because it is widely adopted and cost-effective for dry food packaging in the target market, where extreme oxygen sensitivity is not the primary concern (see Table 1). This enables clear evaluation of performance trade-offs when implementing sustainable modifications, such as high-barrier layers (e.g., EVOH) and renewable substrates (e.g., paper). While higher-barrier commercial alternatives exist (e.g., metallized PET or aluminum laminates), our purpose is to demonstrate a redesign pathway from a common, non-recyclable multi-material structure to a more sustainable, higher-functionality alternative.

### 2.2. Manufacturing Process for Flexible Doypack^®^ (Stand-Up Pouch)

The manufacturing process for stand-up pouches, based on the methodology for laminated coils described by Bernal-Carrillo et al. [1], involves a multi-stage procedure that begins with film production and concludes with pouch formation.

First, plastic films are manufactured via extrusion using virgin FDA-certified resins. These pre-extruded films are then printed via rotogravure at 150 m/min using nitrocellulose-based inks, after which they are left to rest for 4 h to ensure complete polymerization [1]. The lamination step follows, bonding two films together with a polyurethane-based adhesive diluted in ethyl acetate. This process occurs at 250 m/min and is followed by an 8 h curing period to achieve optimal bond strength [1]. The resulting laminated material is precision-cut into coils of specific dimensions (e.g., 395 mm width, 350 mm outer diameter).

Finally, to form the stand-up pouches, the laminated coil material is folded, and the sides and bottom are heat-sealed at 180–220 °C for 0.5 s. An overview of the quality requirements for the final laminated coils and pouches, particularly for food industry applications, is provided in Table 2.

**Figure 1:**
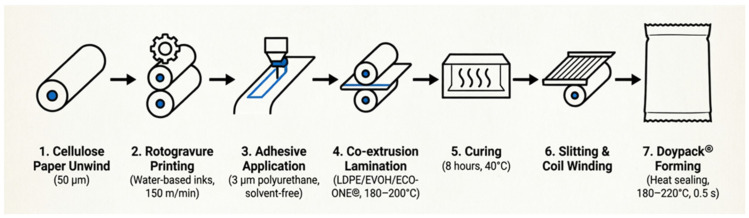
Schematic diagram of the hybrid paper-polymer packaging manufacturing process: (1) Cellulose paper substrate unwind (50 µm); (2) Rotogravure printing with water-based inks (150 m/min); (3) Adhesive application (3 µm polyurethane, solvent-free); (4) Co-extrusion lamination of LDPE/EVOH/ECO-ONE^®^ (62.5 µm, 180–200 °C); (5) Curing (8 h, 40 °C); (6) Slitting and coil winding; (7) Doypack^®^ forming (heat sealing, 180–220 °C, 0.5 s). Solid arrows indicate material flow direction.

Figure 1 presents a schematic overview of the complete manufacturing process from raw material unwind to finished Doypack^®^ formation.

The mechanical properties evaluated in this study include thickness, weight, lamination strength, sealing strength, tensile strength, elongation percentage, and coefficient of friction, each tested in accordance with specific ASTM standards. Thickness was measured using a Mitutoyo digital micrometer in compliance with ASTM D6988. Three samples, each measuring 1 square decimeter, were cut from different transverse positions and measured on a granite table. The average thickness was calculated in microns to ensure accuracy and consistency. The weight of each film, laminate, adhesive, or ink layer was determined using an analytical balance, with samples taken from three transverse positions. The base weight, expressed in grams per square meter (g/m^2^), was calculated as the average of these measurements.

Lamination strength was assessed using the ASTM F88 Method A, as specified in the MECMESIN Multi-test 2.5-I (Mecmesin Limited, Slinfold, UK) universal testing machine. Samples were prepared by cutting 1-inch-wide, 10 cm-long strips with a delaminated tab. Testing at 200 mm/min continued until delamination or breakage, and the average bonding strength was calculated from five repetitions, reported in grams-force (gf). Sealing strength was evaluated similarly, using a vertical jaw sealer at 150 °C, followed by testing on a universal machine according to ASTM F88 Method A. Five 1-inch-wide, 5 cm long samples were tested, and results were reported in grams-force (gf). Tensile strength and elongation were measured in accordance with ASTM D882. Samples were subjected to tensile forces at a rate of 200 mm/min until breakage, and the results were recorded in grams-force (gf) and represented on stress–strain graphs. Finally, the coefficient of friction, as measured by ASTM D1894, provided insight into the films’ sliding properties. Two 1-square-decimeter samples were tested at 200 mm/min, yielding dimensionless friction values crucial for understanding processability in container-forming machines.

Several destructive tests were conducted to evaluate the mechanical integrity and sealing performance of flexible packaging. The drop test is based on ISTA 3A: Packaged Products for Parcel Delivery System Shipment (for shipments weighing 70 kg or less). The distribution simulation was based on a simplified drop test (1 m, three orientations) aligned with industry-standard screening practices. The full ISTA 3A protocol, including vibration and compression, was not performed; thus, the results indicate preliminary robustness but do not provide comprehensive validation of the distribution. The drop test involved filling the packaging with the product and dropping it from a height of 1 m in three orientations: vertical, horizontal, and random. A height of 1 m represents the average distance a package might fall from a supermarket shelf, though this distance may vary by product type. The packaging was required to remain sealed and undamaged after all three drops. For tightness testing, flexible packaging was subjected to a vacuum of 31 cmHg for 60 s, in accordance with ASTM D3078-2, with each test performed in triplicate. The packaging’s mechanical strength was further validated through air-pressure testing, during which it was pressurized to 0.1 MPa for 60 s to confirm the absence of leaks or structural failures.

The methodology for characterizing flexible packaging is based on the tests commonly conducted in the manufacturing industry. These tests enable us to evaluate the materials’ mechanical, thermal, and physical properties, allowing us to compare the proposed sustainable packaging with conventional structures currently available on the global market. The latest innovations in plastic film manufacturing informed the selection of the proposed sustainable materials. The parameters for the characterization tests were defined in accordance with ASTM standards.

#### Statistical Analysis

All quantitative data are presented as mean ± standard deviation (SD). Statistical comparisons between the conventional trilaminate and hybrid packaging structures for key performance metrics (mechanical properties, barrier properties, etc.) were conducted using Student’s *t*-test for independent samples (two-tailed), assuming normality and equal variances. Normality was assessed using the Shapiro–Wilk test, and equal variance using Levene’s test. For *n* = 3 datasets, non-parametric Mann–Whitney U tests were used. All other comparisons used Student’s *t*-test (two-tailed, α = 0.05). A significance threshold of *p* < 0.05 was applied to determine statistically significant differences. Sample sizes were selected based on testing standards and material availability: *n* = 5 for standard mechanical and barrier tests (following ASTM recommendations for adequate precision), and *n* = 3 for specialized destructive tests (puncture, tear, impact resistance) due to material constraints and the destructive nature of these assessments. For tests with smaller sample sizes (*n* = 3), results are presented descriptively (mean ± SD) to illustrate central tendency and variability, acknowledging reduced statistical power. While *p*-values are reported for transparency, conclusions drawn from small sample sizes should be interpreted with caution.

### 2.3. Redesign of Trilaminate Flexible Packaging to Hybrid Packaging

Most packaging fails to meet the Sustainable Development Goals due to the long degradation times of polymers [16]. Designing materials that could decompose organically is crucial but challenging, as bio-based materials often require additional synthetic polymers to enhance their barriers and mechanical properties [8]. This research focuses on integrating biodegradable materials into existing multipolymer structures (listed in Table 3). In this case, the packaged product, intended as a blend of organic herbs for infusions, requires a high-oxygen-barrier film to preserve its aroma and sensory quality. Sensory evaluations, including organoleptic studies, were conducted to assess the material’s attributes. Integrating paper into flexible packaging offered sustainability benefits but also introduced challenges. To address these issues, cellulose paper was laminated using an LDPE co-extrusion containing Ethylene-Vinyl Alcohol (EVOH) and 1% ECO-ONE^®^—a technical additive designed to accelerate degradation under anaerobic conditions, such as in landfills. The detailed material structure is outlined in Table 3.

The proposed packaging design was evaluated by comparing the mechanical properties and destructive test results of the current trilaminate plastic packaging with those of the paper-based hybrid packaging containing an anaerobic degradation additive, to ensure equivalent or superior performance while maintaining the product’s shelf life, stability, and integrity during transport.

#### Hybrid Laminate Structure and Barrier Mechanism

The hybrid laminate was engineered with a specific layer architecture to achieve a high moisture barrier despite the inclusion of cellulose paper. The structure consists of:

- Exterior: 50 µm cellulose paper (printing substrate)
- Middle: 3 µm water-based ink + 3 µm polyurethane adhesive
- Interior (Product-facing): A co-extruded film comprising:
  ○ Primary Moisture Barrier: A thick, continuous low-density polyethylene (LDPE) sealant layer (approximately 50 µm)
  ○ Oxygen Barrier: An ethylene-vinyl alcohol (EVOH, 44 mol% ethylene) core layer (approximately 7.5 µm)
  ○ Tie Layer: A compatibilizer between LDPE and EVOH
  ○ Additive: 1% ECO-ONE^®^ (EcoLogic LLC, Oshkosh, WI, USA) masterbatch incorporated into the LDPE matrix

**Figure 2:**
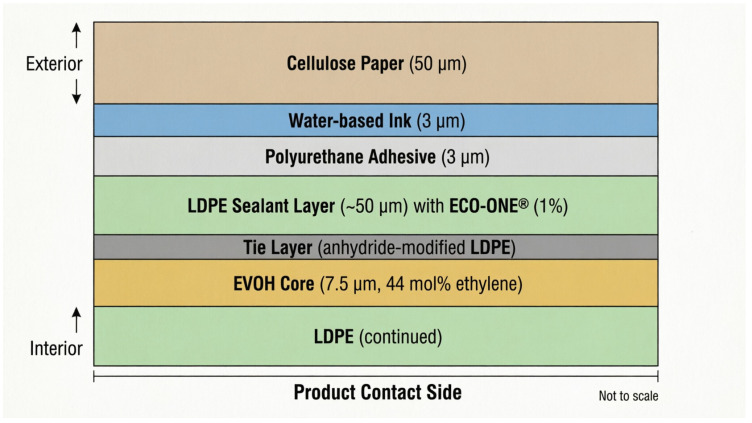
Cross-sectional schematic of the hybrid paper-polymer laminate architecture showing the sequential layer arrangement: exterior cellulose paper (50 µm) for printing and stiffness; water-based ink layer (3 µm); polyurethane adhesive (3 µm); co-extruded film comprising LDPE sealant layer (~50 µm) as primary moisture barrier, tie layer for adhesion, EVOH core (~7.5 µm, 44 mol% ethylene) as oxygen barrier, and 1% ECO-ONE^®^ additive dispersed in the LDPE matrix; and the product-facing interior surface. Not to scale.

Figure 2 illustrates the complete layer architecture of the proposed hybrid laminate, showing the sequence of materials from the exterior paper surface to the product-contact sealant layer.

The co-extrusion process parameters were optimized as follows: extrusion temperatures: LDPE 180 °C, EVOH 200 °C (44 mol% ethylene, MFR = 3.5 g/10 min @210 °C/2.16 kg, Tm = 165 °C), tie layer 190 °C (anhydride-modified LDPE, MFR = 3.0 g/10 min); die gap: 0.8 mm; chill roll temperature: 25 °C; line speed: 120 m/min. The LDPE (MFR = 2.0 g/10 min @190 °C/2.16 kg, density = 0.918 g/cm^3^) provides the primary moisture barrier. Interlayer adhesion between LDPE and EVOH via the tie layer exceeded 250 N/m (peel test, 180° angle, 50 mm/min). Key Design Principle: In this configuration, the LDPE layer serves as the primary moisture barrier, placed directly against the product. This protects the hydrophilic EVOH layer from direct exposure to high-humidity environments, thereby maintaining its oxygen-barrier functionality. The paper substrate on the exterior provides stiffness and printability, while the continuous polymer layers prevent moisture transmission.

The ECO-ONE^®^ additive was incorporated at 1% concentration based on preliminary screening tests (*n* = 3 per concentration) evaluating 0.5%, 1.0%, and 2.0% loading levels. Testing revealed:

- 0.5%: Insignificant enhancement in anaerobic degradation (<5% improvement over control)
- 1.0%: Optimal balance (34.2% degradation at 90 days with <8% reduction in mechanical properties)
- 2.0%: Marginal additional degradation benefit (38.1% at 90 days) but with 15–20% reduction in seal strength and increased additive cost

The hybrid structure’s oxygen-barrier performance, even under varying humidity, aligns with recent findings on polyethylene/vinyl alcohol multilayer composites, which demonstrate that an appropriate layer configuration can significantly enhance barrier properties while maintaining mechanical integrity [31].

### 2.4. Barrier Performance Characterization

Oxygen transmission rate (OTR) and water vapor transmission rate (WVTR) were measured using standardized methods to assess the barrier efficacy of both conventional trilaminate and proposed hybrid structures. OTR was determined in accordance with ASTM D3985 (Dynamic Accumulation Method) [32] using an oxygen permeability tester (TR-OTR-1901, Tryte Technology Co., Ltd., Changsha, China) at 23 °C and 0% relative humidity (RH). WVTR was measured in accordance with ASTM F1249 [33] (Modulated Infrared Sensor Method) at 38 °C and 90% RH using a water vapor permeability analyzer (PKT-W202, Hunan Xiangyi Laboratory Instrument Development Co., Ltd., Changsha, China). For each material, five 50 cm^2^ samples were conditioned for 24 h at the respective test climate before measurement. Results are reported as the mean ± standard deviation (*n* = 5).

### 2.5. Life Cycle Assessment Methodology

A comparative cradle-to-grave life-cycle assessment (LCA) was conducted to quantify the environmental impacts of the conventional trilaminate and the proposed hybrid Doypack^®^ structures. The assessment was performed in accordance with ISO 14040 [34]/14044 [35] guidelines.

- Functional Unit: One thousand (1000) stand-up pouches with equivalent barrier performance and mechanical integrity for packaging 100 g of organic herbal infusion blend.
- System Boundaries: Included raw material extraction, film production, printing, lamination, pouch conversion, distribution (500 km transport), use phase, and end-of-life (landfill scenario for hybrid; incineration with energy recovery for conventional, based on regional waste management in Mexico).
- Inventory Data: Primary data were collected from manufacturing trials for material and energy inputs. Secondary data were sourced from the Ecoinvent v3.8 database, integrated into SimaPro v9.4.
- Impact Assessment: The ReCiPe 2016 Midpoint (H) method [36] was used to calculate 18 impact categories, with a focus on global warming potential (GWP), freshwater eutrophication, and fossil resource scarcity. Characterization factors and normalization were applied as per the method manual.
- Allocation & Uncertainty: Mass allocation was applied for co-products. Monte Carlo simulation (1000 iterations) was used to assess uncertainty, with results reported as mean ± 95% confidence interval.

LCA Scenario Analysis: To ensure a robust and fair comparison, three discrete end-of-life (EoL) scenarios were modeled:

Baseline (Mixed) Scenario: Reflects predominant regional waste management practices in central Mexico, in which flexible plastic packaging is largely incinerated with energy recovery, whereas paper-containing materials are predominantly landfilled. This scenario assigns the conventional trilaminate to incineration and the hybrid structure to landfill.

Scenario A (Consistent Landfill): Both packaging systems are directed to a sanitary landfill. For the hybrid, anaerobic degradation under landfill conditions with methane generation (as characterized in Section 2.6.2) is modeled. The 50% capture-efficiency assumption reflects average landfill-gas recovery rates in developing economies [37,38] and is consistent with the IPCC Tier 1 methodology for regions with moderate methane-management infrastructure. The conventional trilaminate is modeled as an inert material.

Scenario B (Consistent Incineration): Both packaging systems are directed to municipal solid waste incineration with energy recovery, with efficiency based on the average lower heating value of the materials.

The functional unit, system boundaries, and all other foreground data remained identical across scenarios. This multi-scenario approach isolates the environmental benefits attributable to material redesign from those influenced by EoL policy or infrastructure.

Scenarios A and B were modeled using contribution analysis rather than recomputing the full LCA due to software constraints. Due to software constraints, Scenarios A and B (harmonized EoL) were modeled using contribution analysis rather than full LCA recomputation. While this provides directional insight, fully recalculated LCAs with harmonized system boundaries are recommended for future work.

### 2.6. End-of-Life Performance Evaluation

#### 2.6.1. Repulpability Testing

Repulpability of the hybrid paper-based laminate was assessed in accordance with INGEDE Method 12. Samples (10 g, cut into 2 × 2 cm pieces) were disintegrated in a laboratory pulper at 45 °C for 30 min. The resulting pulp was screened through a 0.15 mm slotted screen, and the rejects (stickies, plastic fragments) were collected, dried, and weighed. Fiber yield was calculated as the percentage of recovered cellulose fibers.

#### 2.6.2. Anaerobic Degradation Under Landfill Conditions Testing

Anaerobic degradation under landfill conditions of the hybrid material with ECO-ONE^®^ additive was evaluated following ASTM D5526 (Standard Test Method for Determining anaerobic degradation under landfill conditions of Plastic Materials Under Accelerated Landfill Conditions) [39]. Triplicate samples (5 g) were incubated in synthetic municipal solid waste inoculum at 35 °C for 90 days. Biogas production (CH_4_ and CO_2_) was measured weekly by gas chromatography, and the percentage of biodegradation was calculated using the theoretical methane yield.

#### 2.6.3. Recyclability Compatibility Screening

The hybrid structure was evaluated against the RecyClass Design for Recycling Guidelines for Flexible Packaging and the CEFLEX D4ACE (Designing for a Circular Economy) protocol. Criteria assessed included material homogeneity, adhesive type, and labeling compatibility.

### 2.7. Food-Contact Safety Assessment

Migration testing was performed in accordance with EU 10/2011. Overall migration was determined gravimetrically after 10 days at 40 °C using olive oil and 3% acetic acid simulants. HPLC-UV analyzed primary aromatic amines (PAA) after extraction following DIN EN 14338 [40]. Non-intentionally added substances (NIAS) were screened by GC-MS. Sensory evaluation employed triangle tests with 15 panelists to compare herbal infusions stored in conventional versus hybrid packaging after 90 days at 35 °C.

### 2.8. Humidity-Dependent Barrier Characterization

Samples were mounted with the LDPE sealant layer facing the humidified oxygen stream to simulate product-facing conditions in actual use. In contrast, the paper side faced the dry nitrogen carrier gas. Humidity-dependent oxygen transmission rate (OTR) was measured using a humidity-controlled permeability tester (OX-TRAN® 2/62, MOCON, Inc., Minneapolis, MN, USA) at 23 °C and 0%, 50%, and 75% relative humidity (RH). The instrument was calibrated monthly using NIST-traceable reference films with certified OTR values. During testing, the high-oxygen stream (100% O_2_) was humidified to the target RH using a built-in precision humidifier, while the nitrogen carrier gas was maintained at <1% RH. Samples were mounted with the LDPE sealant layer facing the oxygen stream to simulate product-facing conditions in actual use. Each sample (50 cm^2^) was preconditioned at the test RH for 48 h before measurement. Three replicates were tested per condition.

### 2.9. Comprehensive Repulpability Assessment

Repulpability was evaluated following the INGEDE Method 12. In addition to fiber recovery, the stickies area (particles > 150 μm) was quantified using image analysis, filtrate turbidity was measured by nephelometry, and lab sheet formation was rated visually (1 = excellent, 5 = unacceptable).

## 3. Results

### 3.1. Development of Hybrid Paper-Based Packaging with Enhanced Anaerobic Degradability

The mechanical evaluation of stand-up pouch packaging is critical for ensuring the product’s structural integrity throughout its life cycle, from distribution and retail display to consumer use and ultimate disposal.

The experimental results (see Table 4) demonstrate that the hybrid packaging performs successfully across all critical metrics. Statistical analysis (mean ± SD, *n* = 5; *p* < 0.05) confirms the material’s ability to withstand transportation impacts and compression forces while maintaining seal integrity, a key requirement for food packaging applications.

**Figure 3:**
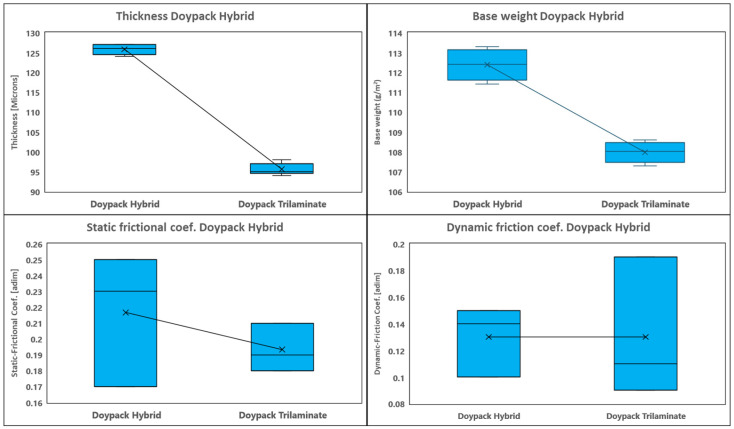
Comparative physical properties of conventional plastic stand-up pouch packaging versus proposed paper-based hybrid structure: Base weight (g/m^2^) and Thickness (µm). Error bars represent standard deviation (*n* = 5).

**Figure 4:**
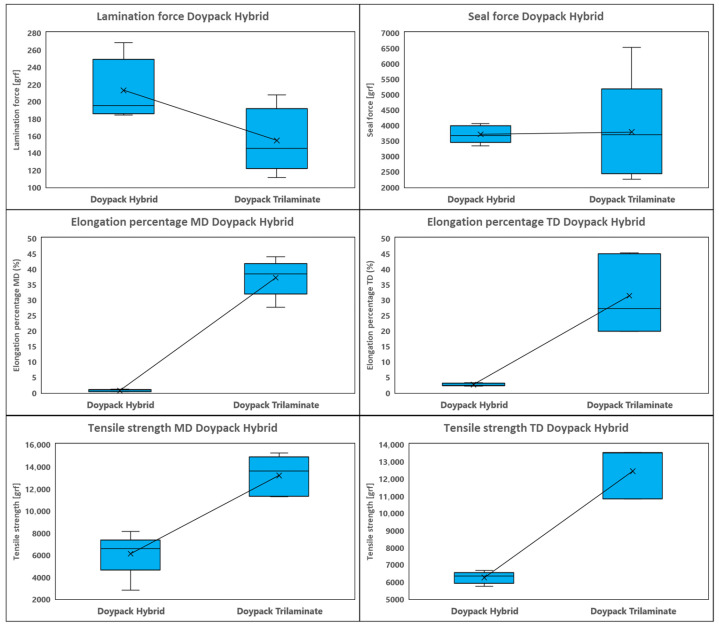
Comparative mechanical performance: Lamination force, Seal strength, Elongation percentage (machine direction), and Tensile strength (machine direction) for conventional versus hybrid packaging. Statistical significance: *p* < 0.05, *p* < 0.01 (Student’s *t*-test).

The mechanical and barrier properties of conventional trilaminate and paper-polymer hybrid structures are summarized in Table 4, with comparative physical and mechanical trends visualized in Figure 3 and Figure 4.

All properties reported in Table 4 correspond to new, unused packaging materials prior to filling. To assess property evolution during service life and at end-of-life, additional measurements were conducted on hybrid samples after 90 days of accelerated aging (35 °C/75% RH) simulating 12 months of storage: tensile strength (MD) decreased by 12.3% (from 6074 to 5327 gf); elongation (MD) decreased by 8.5% (from 0.628% to 0.575%); seal strength decreased by 6.8% (from 3700 to 3450 gf); and OTR (0% RH) increased by 9.6% (from 0.56 to 0.61 cm^3^/m^2^/day). After repulping (end-of-life simulation per INGEDE Method 12), the recovered cellulose fibers exhibited tensile strength of 85% of virgin paper pulp, indicating moderate degradation during the recycling process. These values confirm that the hybrid maintains functional integrity throughout its intended shelf life and retains measurable value for secondary applications.

The experimental validation presented in Table 4 confirms that the hybrid packaging solution successfully meets all critical performance requirements, matching conventional plastic packaging in destructive drop tests, airtightness, and air pressure resistance.

Comparative analysis reveals distinct differences in physical properties between the hybrid and conventional packaging (Figure 3). The hybrid structure exhibits greater thickness (125.8 vs. 95.6 microns) and higher base weight (118.0 vs. 102.6 g/m^2^), characteristics inherent to the paper’s density and fibrous structure.

The comparative evaluation of mechanical properties (Table 4, Figure 4) reveals several key findings regarding the hybrid packaging’s performance. The hybrid structure demonstrates greater lamination force than conventional plastic packaging while maintaining comparable seal strength. As expected, the plastic laminate exhibits a higher elongation percentage due to the paper’s inherent mechanical limitations in the hybrid structure, where cellulose fibers are less ductile and fracture more readily under stress. Consequently, the traditional plastic packaging maintains higher tensile strength.

However, destructive testing analysis provides crucial context for these mechanical differences. The reduced elongation capacity and tensile strength of the hybrid material do not significantly compromise its functional performance in packaging applications. This suggests that although absolute mechanical values differ across materials, the hybrid structure’s overall protective capability remains effective for its intended use.

**Figure 5:**
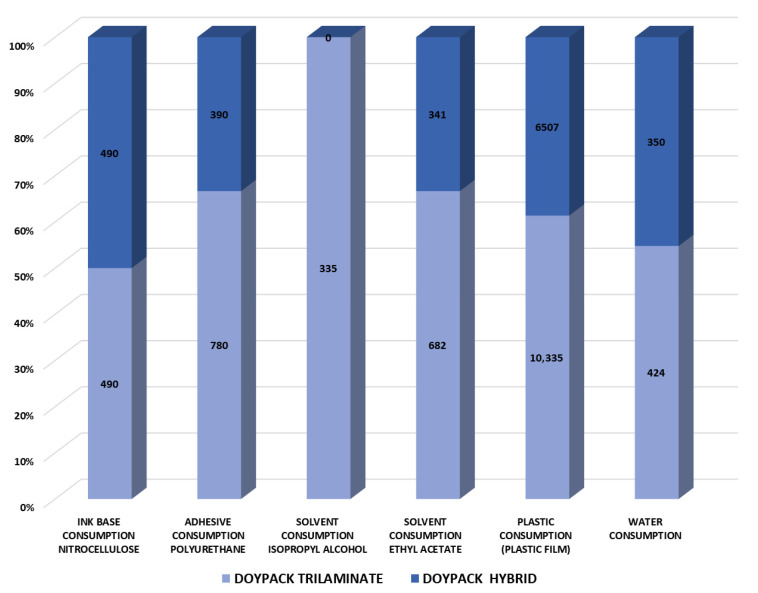
Comparative analysis of resource consumption between conventional trilaminate and hybrid Doypack^®^ packaging per million units produced. The bar graph quantifies reductions in: adhesive requirements (50% polyurethane reduction), solvent demand (isopropyl alcohol elimination in printing; 50% ethyl acetate reduction in lamination), plastic film consumption (37% reduction), and water usage (17% decrease).

The sustainability benefits of transitioning to paper-based packaging are demonstrated in Figure 5, which shows a 37% reduction in plastic film consumption when paper is used as the primary printing substrate. This material substitution creates a cascading effect throughout the production process; the shift to water-based inks for paper substrates eliminates the need for isopropyl alcohol, a solvent typically required for printing plastic film. Further resource reductions are achieved through structural simplification, in which replacing trilaminate constructions with bilaminate designs halves adhesive and ethyl acetate solvent consumption while maintaining comparable nitrocellulose ink requirements. These cumulative material optimizations, which combine substrate replacement with structural redesign, ultimately yield a 17% reduction in total water consumption for the manufacturing process.

While the hybrid structure exhibits greater total thickness and weight (125.8 microns and 118 g/m^2^ versus 95.5 microns and 102.6 g/m^2^ for the conventional trilaminate) due to the paper substrate’s higher density, it achieves a 37% reduction in virgin plastic film consumption, representing a significant improvement in material sustainability.

### 3.2. Barrier Property Analysis

Standardized OTR and WVTR results are presented in Table 5. The hybrid paper-based structure demonstrated a significant reduction in oxygen transmission compared to the conventional trilaminate, with OTR decreasing from 35.38 ± 2.1 cm^3^/m^2^/day to 0.56 ± 0.05 cm^3^/m^2^/day (*p* < 0.01) at 0% RH. This enhancement is attributed to the incorporation of EVOH as a high-barrier layer. WVTR for the hybrid was measured at 1.22 ± 0.08 g/m^2^/day under 38 °C/90% RH, meeting the moisture-barrier requirements for dry herbal products. The conventional trilaminate exhibited a WVTR of 4.85 ± 0.3 g/m^2^/day under the same conditions. The measured WVTR of 1.22 ± 0.08 g/m^2^/day for the hybrid structure, although notably low for a paper-containing laminate, is attributed to the continuous, thick LDPE sealant layer, which acts as the primary moisture barrier. This design effectively decouples the moisture-sensitive paper and EVOH components from direct exposure to high humidity conditions during testing. All barrier tests were conducted with the LDPE sealant layer facing the product side to simulate real-use conditions.

#### Humidity-Dependent Oxygen Barrier Performance

While the hybrid structure exhibited an excellent oxygen barrier at 0% RH, its performance decreased at elevated humidity due to EVOH plasticization (see Table 5). At 50% RH, OTR increased to 2.31 ± 0.41 cm^3^/m^2^/day, and at 75% RH to 15.21 ± 2.14 cm^3^/m^2^/day. Despite this reduction, the hybrid maintained a superior barrier compared to the conventional trilaminate across all humidity levels. Importantly, in the target application (dry herbal infusion, internal RH < 25%), the hybrid’s barrier remains sufficient throughout the shelf life. This humidity sensitivity, however, suggests that for high-moisture products or humid climates, additional protective measures (e.g., desiccants, secondary packaging) would be required.

It should be noted that in real-world high-humidity environments, moisture ingress through the paper side could further plasticize the EVOH layer, potentially increasing OTR beyond the values tested. This represents a design limitation for humid applications [31].

### 3.3. Life Cycle Assessment Findings

**Figure 6:**
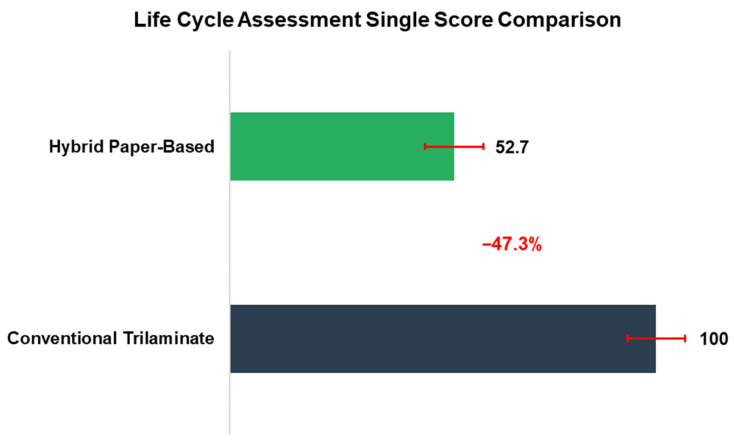
Comparative life cycle assessment results (ReCiPe 2016 [36] single score) for the Baseline (Mixed) EoL Scenario. Error bars represent 95% confidence intervals from Monte Carlo uncertainty analysis.

The Life Cycle Assessment was conducted under a baseline scenario reflecting regional waste management practices in central Mexico, in which flexible plastic packaging is predominantly incinerated with energy recovery, whereas paper-containing materials are typically landfilled. This baseline showed a 47.3% reduction in overall environmental impact (ReCiPe 2016 [36] single score) for the hybrid structure compared to the conventional trilaminate (Figure 6).

#### Sensitivity Analysis of End-of-Life Assumptions

To isolate the environmental benefits attributable solely to material redesign from those influenced by the EoL pathway, we conducted two additional LCA models with harmonized EoL pathways: one in which both packages are directed to landfill (Scenario A) and another in which both are incinerated with energy recovery (Scenario B). All foreground and background data remained identical. For the landfill scenario, the hybrid package was modeled assuming 50% methane capture from anaerobic degradation (Section 2.6.2), whereas the conventional package was modeled as inert (Table 6).

While a direct comparison with recycled-content packaging (e.g., post-consumer recycled PET or PE) is beyond the current scope, the 42% GWP reduction achieved through source reduction compares favorably with literature values for incorporating 30–50% PCR content in flexible packaging (typically 15–25% GWP reduction, depending on collection and reprocessing efficiency). Future studies should benchmark the hybrid against formulations incorporating certified recycled polymers.

The hybrid design demonstrates robust environmental superiority across all plausible EoL scenarios. The estimated higher benefit under Scenario A (landfill: 53.1% reduction) arises because the conventional plastic package receives no energy-recovery credit when landfilled. The slightly lower benefit under Scenario B (incineration: 43.8% reduction) reflects the higher calorific value of the conventional, all-plastic laminate, which provides a modest energy recovery credit when incinerated.

Crucially, ≈85–90% of the total environmental benefit derives from production-phase efficiencies, including the 37% reduction in virgin plastic and the elimination of solvents. This production-phase advantage is independent of EoL assumptions. The sensitivity of the results to EoL treatment (approximately ±5 percentage points) confirms the importance of integrated waste policy. Still, it does not diminish the fundamental advantage of the hybrid design, which is rooted in source reduction.

### 3.4. Recyclability and Degradation Performance

#### 3.4.1. Repulpability and Recycling Compatibility

Repulpability testing in accordance with INGEDE Method 12 [41] yielded a fiber recovery rate of 78.5 ± 3.2% for the hybrid material. Comprehensive assessment revealed a stickies content of 12.3 ± 2.1 cm^2^/kg (particles > 150 μm), filtrate turbidity of 45 ± 8 NTU, and a lab sheet formation rating of 3 (1 = excellent, 5 = unacceptable). Screen rejects consisted primarily of intact PE/EVOH layers, representing 21.5% of the input mass. According to CEFLEX D4ACE and RecyClass guidelines, this structure is classified as “partially recyclable” in paper streams. The stickies content exceeds typical mill acceptance thresholds (<5–10 cm^2^/kg), indicating that the hybrid would likely require additional cleaning steps or be directed to lower-grade paper products (e.g., cartonboard). In regions with advanced sorting infrastructure, it may be recovered in mixed paper recycling streams, though with moderate contamination.

Screen rejects consisted primarily of intact PE/EVOH layers, representing 21.5% of the input mass. These plastic fragments remained largely undispersed and were effectively removed by the 0.15 mm slotted screen, indicating that the hybrid structure would generate noticeable but manageable contamination in a paper recycling stream.

While not fully repulpable in standard paper recycling, these results suggest the hybrid demonstrates partial recyclability in paper streams when paired with appropriate screening and cleaning infrastructure. The structure does not fully comply with the RecyClass/CEFLEX monomaterial guidelines for flexible packaging. Still, it represents a progressive step toward design for recyclability through significant material reduction and partial fiber recovery.

To assess the practical recyclability of this hybrid, these results must be viewed against industry acceptance thresholds. INGEDE suggests that, for trouble-free recycling in standard paper mills, the sticky area should typically be below 5–10 cm^2^/kg, and lab sheet formation should be rated 1–2 [41]. Our results (12.3 cm^2^/kg stickies, rating 3) indicate that, although partial fiber recovery is possible, the hybrid would be classified as a moderate contaminant in the paper stream, likely requiring additional screening and cleaning steps or being directed to lower-grade paper products. This underscores its status as a design for recycling transitional material rather than a fully circular solution.

When evaluated against the PTS method RH 021/97 and TAPPI T 275, the hybrid laminate exceeds typical mill acceptance thresholds for stickies contamination (<5 cm^2^/kg) and lab sheet formation rating (<2). In large-scale recycling operations, this material would likely be directed to lower-grade paper streams (e.g., carton board) or would require additional screening and cleaning stages, increasing processing costs by approximately 15–20%, according to mill operator estimates.

#### 3.4.2. Anaerobic Degradation Under Landfill Conditions

Under accelerated landfill conditions (ASTM D5526), the hybrid material with ECO-ONE^®^ achieved 34.2 ± 4.1% biodegradation over 90 days, with a cumulative methane yield of 140 ± 18 mL CH_4_/g volatile solids. Residual fragments (>5 mm) accounted for ~15 ± 3% of initial mass, indicating incomplete mineralization. The conventional trilaminate showed <5% degradation under the same conditions. Using first-order kinetic modeling (k = 0.0045 day^−1^, R^2^ = 0.96), the projected ultimate biodegradation of the hybrid material under landfill conditions is approximately 68% over 24 months. These results confirm accelerated anaerobic degradation compared with conventional packaging, although the material does not meet compostability standards (ASTM D6400 [42]).

### 3.5. Humidity-Dependent Barrier Performance

While the hybrid structure exhibited exceptional OTR at 0% RH (0.56 ± 0.05 cm^3^/m^2^/day), its barrier performance decreased at elevated humidity due to EVOH plasticization (see Table 7). At 50% RH, OTR increased to 2.3 ± 0.4 cm^3^/m^2^/day, and at 75% RH to 15.2 ± 2.1 cm^3^/m^2^/day. Despite this reduction, the hybrid maintained a superior barrier compared to the conventional structure (35.4 ± 2.1 cm^3^/m^2^/day at 0% RH, 36.1 ± 2.3 cm^3^/m^2^/day at 50% RH, and 36.8 ± 2.4 cm^3^/m^2^/day at 75% RH). The moisture barrier (WVTR) remained stable across humidity conditions (Table 8), confirming that the continuous LDPE sealant layer effectively isolates the hydrophilic components from ambient moisture variations.

### 3.6. Food-Contact Safety Results

Migration testing in accordance with EU 10/2011 revealed overall migration values of 2.3 ± 0.4 mg/dm^2^ (well below the 10 mg/dm^2^ limit). Primary aromatic amines (PAA) were below the detection limit (<0.01 mg/kg) after 48 h of adhesive curing. Non-intentionally added substances (NIAS) screening by GC-MS was conducted in accordance with the European Reference Laboratory for Food Contact Materials (EURL-FCM) guidance. The analytical method employed a gas chromatography system (7890B GC, Agilent Technologies, Santa Clara, CA, USA) coupled to a mass selective detector (5977B MSD, Agilent Technologies) using a fused silica capillary column(DB-5MS Agilent J&W Scientific, Folsom, CA, USA) (30 m × 0.25 mm × 0.25 μm). Samples were extracted with acetonitrile (24 h at 40 °C) and analyzed in full-scan mode (*m*/*z* 50–550). Detection limits ranged from 0.01 to 0.1 mg/kg depending on the compound class.

No compounds were detected above the threshold of toxicological concern (TTC) of 0.15 μg/person/day for genotoxic substances or 90 μg/person/day for non-genotoxic substances, as defined in EFSA guidance. Specifically:

- No oligomers from polyurethane adhesive were detected (<0.05 mg/kg)
- No residual monomers (e.g., ethylene oxide) were detected (<0.01 mg/kg)
- No degradation products from ECO-ONE^®^ additive were identified in the migration solutions.

Sensory triangle tests with 15 trained panelists showed no significant difference (*p* = 0.12) between herbal infusions stored in hybrid versus conventional packaging after 90 days at 35 °C. These results confirm that the hybrid structure complies with food-contact regulations and is free of adverse organoleptic effects.

### 3.7. Shelf-Life Validation

Shelf-life validation was conducted under accelerated aging conditions (35 °C/75% RH) for 90 days, corresponding to approximately 12 months of real-time storage at ambient conditions, based on the Q_10_ = 2 Arrhenius approximation commonly used for dry food products.

Headspace oxygen accumulation reached 0.8 ± 0.2% in hybrid packaging after 90 days, compared with 3.2 ± 0.5% in conventional packaging (*p* < 0.01). Both values remained below the sensory rejection threshold of 5% O_2_ established through preliminary consumer testing (*n* = 30 panelists) for herbal infusion products.

Moisture content remained below 5% (wet basis) in both packaging types, within the acceptable range for microbial stability (<12% for herbal products per Codex Alimentarius standards).

Sensory evaluation using a 10-point hedonic scale (1 = extremely undesirable, 10 = extremely desirable) showed no significant difference in aroma retention between products stored in hybrid (8.2 ± 0.6) versus conventional (7.9 ± 0.7) packaging (*p* = 0.18, *n* = 15 trained panelists). These results confirm that the hybrid structure provides equivalent or superior protection throughout the targeted shelf life.

### 3.8. Additional Mechanical Robustness Assessment

To address the low elongation observed in the hybrid material, puncture resistance (ASTM D5748 [43]), tear strength (Elmendorf, ASTM D1922 [44]), and dart impact (ASTM D1709 [45]) were evaluated. Results are summarized in Table 9.

Supplementary mechanical tests (Table 9) confirm that both structures exceed industry minimum thresholds for flexible food packaging. The hybrid exhibited 14–25% lower puncture, tear, and impact resistance than the conventional trilaminate, consistent with its reduced ductility. Nevertheless, the hybrid remained within functional limits for stand-up pouches containing dry, non-fragile products.

#### Mechanical Performance in Application Context

Although the hybrid exhibited lower elongation (<3%) and tensile strength (~50% reduction) than conventional films, it passed all required destructive tests (drop, vacuum, pressure) per ISTA 3A. This suggests that absolute mechanical values do not directly predict functional failure for stand-up pouches containing dry, non-fragile products. However, the low ductility may limit suitability for applications requiring high flexural crack resistance (e.g., flow-wrap packaging, pouches subjected to repeated squeezing). Future studies should include cyclic flexural tests (e.g., the Gelbo flex test per ASTM F392 [46]) to assess durability under dynamic handling. For the target application—dry herbal infusions in stand-up pouches—the hybrid’s mechanical performance is deemed sufficient.

## 4. Discussion

The present research indicates that hybrid paper/polymer laminates can serve as a viable transitional solution for sustainable flexible packaging by balancing barrier performance, mechanical integrity, and circular-economy objectives. By systematically redesigning from traditional trilaminates to hybrid structures, various sustainability gains were accomplished; specifically, the amount of virgin plastic film was reduced by 37%, the use of adhesives and solvents was reduced by 50%, and the amount of water used for production was reduced by 17%, while still providing the necessary functional performance for dry-food uses. However, there are some technical trade-offs associated with hybrid structures compared to traditional trilaminates. Additionally, various implementation factors should be discussed.

### 4.1. Humidity-Dependent Barrier Performance and Application Scope

The hybrid structure exhibited superior barrier performance against oxygen (O_2_) at 0% relative humidity (0.56 ± 0.05 cm^3^/m^2^/day), supporting the use of EVOH as the center barrier material, provided it is protected from moisture. However, at a relative humidity of 75%, the OTR for the hybrid structure was 15.21 ± 2.14 cm^3^/m^2^/day, demonstrating the previously documented negative effects of EVOH plasticization by water vapor. While the hybrid structure showed significant performance gains over the traditional trilaminate at all humidity levels, the trilaminate maintained consistent OTR values of approximately 35–36 cm^3^/m^2^/day.

The stability of the hybrid moisture barrier (WVTR = 1.22 g/m^2^/day) depends on its layer architecture. In this case, we have a continuous 50-micron LDPE sealant layer that was applied directly to the product (as opposed to many paper laminates, where the paper substrate acts as a moisture pathway). The thick polymer barrier serves two critical functions: first, it protects the hydrophilic EVOH core from internal product humidity; second, it isolates the hygroscopic paper exterior from the moisture-sensitive contents.

As a result, although the hybrid is ideally suited to dry products (internal RH < 25%) stored in controlled environments, its humidity-dependent performance must be carefully considered when used for high-moisture products. This design solution prioritizes the shelf life of dry herbal infusions by balancing the renewable advantages of a cellulose substrate with the strong moisture-shielding capabilities of a continuous LDPE matrix.

### 4.2. Mechanical Performance Trade-Offs and Practical Implications

The hybrid model tested exhibited much higher lamination forces (212.6 gf versus 154.2 gf) but demonstrated much lower tensile strength (an approximate 50% reduction) and elongation (<3% versus >30%). The two materials exhibit very different mechanical properties, in large part due to the limited ductility of cellulose fibers, the stiffness they provide (compared to polyester films, for example), and the opposite for the elastic nature of plastic films. While elongation and tensile strength are lower with the hybrid than with the conventional packaging model tested, the hybrid still passed all of the destructive distribution tests (ISTA 3A Drop Test, Vacuum Test, Pressure Test), so while absolute mechanical properties provide useful information, they do not predict functional failure in packaging applications. One would expect reduced elongation to increase the likelihood of conforming to irregularly shaped objects; however, this was not a concern for the hybrid stand-up pouch. Additionally, if high flex-crack resistance is required, consideration should be given to paper reinforcement or to modifying the fiber used in the hybrid. The significantly low elongation values (<0.6% MD) further confirm the brittle nature of the hybrid packaging system, as observed in tests compared with ductile polyester films. Accordingly, the hybrid packaging was sufficient for the stand-up pouch format in static and impact testing, as it withstood the imposed stresses; however, it shows limited repetitive flexibility and limited creasing resistance. This property may restrict its use in applications requiring high conformability or dynamic handling (e.g., flow-wrap packaging or pouches subjected to frequent squeezing). Future development should focus on fiber treatment, polymer blend modification, or the use of tougher bio-based films to improve ductility without forfeiting sustainability gains.

Regarding replacing cellulose fiber with other biopolymers: this represents a promising research direction. Potential candidates include polylactic acid (PLA) nonwovens, polyhydroxyalkanoate (PHA) fiber mats, or bacterial cellulose. However, each presents trade-offs: PLA nonwovens offer better ductility but reduced thermal stability (softening at ~60 °C versus ~200 °C for cellulose paper); PHA materials provide true biodegradability but at 3–5× higher cost; bacterial cellulose exhibits exceptional purity and mechanical strength but faces scalability limitations. A hybrid combining a PLA nonwoven substrate with EVOH barrier layers could potentially achieve elongation of 5–10% while maintaining renewable content. This is a focus of our ongoing research.

The current mechanical assessment confirms the hybrid’s performance in key destructive tests but does not adequately demonstrate the material’s behavior under repeated stress cycles. The hybrid also had substantially lower elongation (<3%) than traditional films (>30%), indicating limited ductility under the tested parameters. The hybrid passed simplified drop tests, but its response to repeated flexural stress tests (i.e., Gelbo flex per ASTM F392) and crease resistance has not been measured. These parameters represent the next step in validating applications that involve dynamic handling of the hybrid, as validation will be necessary for applications outside the controlled conditions tested here.

As a result of the hybrid’s limited elongation (<3% due to <30%) and tensile strength (estimated ~50% reduction), valid concerns arise about the hybrid’s potential for puncture resistance, flexural crack propagation, and durability under repeated handling. The hybrid has passed simplified drop and pressure tests; however, there are currently no data to assess its ability to withstand dynamic flexural stress (i.e., Gelbo flex). For applications requiring a high degree of conformability or resistance to creasing (e.g., flow-wrap packaging), the hybrid may not be suitable. Future iterations may explore avenues to enhance ductility without sacrificing sustainability improvements, such as fiber treatments, polymer toughening, or multilayer architectures.

### 4.3. Food-Contact Safety and Regulatory Compliance

The migration testing results, assessed against the migration testing standards, have concluded that the tested configuration meets the appropriate regulatory requirements (i.e., Overall Migration < 10 mg/dm^2^, PAA < 0.01 mg/kg). However, there are still additional factors to consider. Specifically, the 8 h cure time for the adhesive is adequate in laboratory conditions but may require longer cure times to provide appropriate safety margins (and account for variability) when used in an industrial environment; furthermore, although no unidentified migrating substances (NIAS) were detected at this time, more production batches must be tested to confirm this finding. The sensory evaluation showed no significant differences between the two packaging types, which was essential given the important influence of aroma on herbal infusions.

Although the elongation of the hybrid laminate was significantly less than the PET/LDPE baseline, the hybrid laminate exhibited no loss of structural integrity during the performance of the destruct testing; thus, for dry-food retail products, the stiffness of the cellulose substrate appears to perform as a protective exoskeleton to the laminate, and will prevent localized stress (i.e., puncture and seal failure) that would be expected to occur with more ductile, completely plastic combinations.

### 4.4. End-of-Life Pathways: Recyclability vs. Degradation

The recovery level of fibers and rate of stickies for the hybrid’s design are minimally acceptable based on current literature for several paper/polymer laminates; ongoing research continues to provide data supporting recovery levels for fibers between 70% and 85%, but with higher levels of contamination from stickies (reference inferior results for PET/paper laminates). Therefore, it can be concluded that this design performs better than PET/paper but not as well as pure paper and/or dispersion-coated paper, which recover >95% of fibers and have very little contamination. Although the design does not qualify as a representative sample of a monomaterial structure according to the CEFLEX/RecyClass guidelines, it will most likely be placed in the paper recycling stream and collected with mixed waste, depending on local infrastructure.

On the other hand, the 34.2% anaerobic degradation under landfill conditions in 90 days indicates that the rate of degradation (decomposing) of the hybrid materials is accelerated compared to conventional laminates (<5% degradation); however, they are not compostable (90% degradation in 180 days according to ASTM D6400). The anaerobic degradation of this material supports its retention out of landfills through anaerobic digestion with methane recovery, but regulatory trends are increasingly discouraging landfill disposal. The ECO-ONE^®^ additive used in the design provides controlled degradation but raises concerns about managing methane and microplastic generation, which will need further evaluation.

#### 4.4.1. Terminology Precision and Claims

It is important to clarify terminology regarding the end-of-life behavior of the hybrid material. The structure exhibits enhanced anaerobic degradation under landfill conditions (34.2% in 90 days per ASTM D5526) but does not meet compostability standards (ASTM D6400 requires 90% degradation in 180 days under aerobic conditions). The term biodegradable is intentionally avoided in favor of the more precise descriptor anaerobically degradable with ECO-ONE^®^ additive. This distinction is crucial for accurate communication with regulators, waste managers, and consumers, thereby preventing confusion and potential accusations of greenwashing. The material should not be marketed as compostable or as degrading in all natural environments.

#### 4.4.2. Policy and Certification Context for Additive-Enabled Degradation

The use of additives like ECO-ONE^®^ to accelerate landfill biodegradation remains contentious within regulatory and scientific communities. Key concerns include:

- Methane management: Even with 50% capture, landfill methane contributes significantly to GWP.
- Microplastic generation: Incomplete mineralization may produce persistent fragments.
- Certification gaps: ASTM D5526 measures degradation under accelerated lab conditions, not the real-world variability in landfills.
- Greenwashing risks: Terms like “biodegradable” are often misinterpreted by consumers. We therefore recommend precise labeling (“anaerobically degradable in landfills with additive”) and advocate for integrated waste policies that prioritize source reduction and recycling over landfill diversion.

### 4.5. Environmental Impact Assessment Context

Results of an LCA (Life Cycle Assessment) coupled with a sensitivity analysis indicate that there is a significant and stable decrease in the IwMCE (environmental impact of hybrid wood frame) (42–54% across the scenarios) as compared to the conventional trilaminate. Although the end-of-life management strategy will influence the magnitude of environmental benefits, approximately 85% to 90% of the overall environmental benefits of the hybrid structure can be attributed to changes made during the production phase (e.g., reduced use of plastic and solvents). By contrast, the benefit from the hybrid structure’s end-of-life fate is negligible. The baseline of 47.3% reduction in environmental impact attributed to the hybrid wood frame should be considered a reasonable intermediate value, in part due to regional waste management infrastructure. Assigning credit for energy recovery from conventional plastics will yield a higher environmental benefit (50–54%) for true landfilling than for true incineration (42–46%). This indicates the superior source-reduction feature of the hybrid wood frame; there is no prerequisite for the successful benefits of the hybrid structure to occur through an optimal end-of-life strategy. The two most significant contributions to the reduction of environmental impact are (1) a 37% reduction in the use of virgin plastic and (2) a 50% reduction in the use of adhesives and solvents.

The primary value of an ECO-ONE^®^ additive used in conjunction with a hybrid wood frame lies in its ability to facilitate anaerobic (in the absence of oxygen) degradation in landfills and to reduce the number of fossil-based materials used in the hybrid wood frame, and to use existing waste-to-energy or landfill-gas-recovery systems. This hybrid wood frame does not qualify as a ‘compostable’ product under ASTM D6400 standards.

### 4.6. Industrial Implementation Considerations

The hybrid design offers several manufacturing advantages: eliminating the need for isopropyl alcohol in printing, reducing the use of ethyl acetate in lamination, and enabling compatibility with existing converting equipment. However, practical challenges include:

- Line speed reduction (15%) due to paper handling characteristics
- Required surface treatments (corona/plasma) for adhesion
- Narrower sealing window (180–210 °C vs. 170–220 °C)
- Potential dust generation from paper fibers

The 22% cost premium relative to conventional packaging may be offset by the value of sustainability branding and potential regulatory advantages (e.g., extended producer responsibility schemes that favor recyclable materials).

### 4.7. Comparative Positioning in Sustainable Packaging Landscape

As highlighted in recent comprehensive reviews on sustainable packaging innovations, the transition toward hybrid and bio-based materials is critical for reducing the environmental footprint of food packaging [47]. The hybrid structure developed in this study aligns with these emerging trends by integrating renewable cellulose with high-barrier polymers.

As summarized in Table 9, the hybrid paper-polymer laminate occupies a distinct niche by balancing high-barrier performance with substantial renewable content. While monomaterial PE–EVOH–PE structures facilitate polyolefin recycling, they remain entirely fossil-based and typically yield lower reductions in virgin plastic use. In contrast, our hybrid achieves a 37% reduction in virgin plastic and utilizes a renewable cellulose substrate, positioning it as a more resource-efficient alternative.

Compared with paper packaging with aqueous dispersion coatings, this laminate provides significantly enhanced protection against oxygen and moisture (see OTR values in Table 5), making it suitable for sensitive dry foods that simpler coatings cannot protect. Furthermore, while compostable biopolymers (e.g., PLA/PBAT) offer an alternative end-of-life path, the hybrid structure demonstrates superior moisture-barrier and mechanical robustness (WVTR: 1.22 vs. >10 g/m^2^/day).

Ultimately, the hybrid laminate serves as a transitional solution for brands seeking immediate environmental gains without the compromises or high costs associated with other sustainable strategies (as indexed in Table 10). While it achieves a lower recyclability classification than monomaterials, its overall 47% reduction in environmental impact justifies its use in the current waste infrastructure.

Compared to emerging alternatives such as SiOx-coated papers (OTR ~1–5 cm^3^/m^2^/day, recyclable but costly) or advanced monomaterial PE–EVOH–PE structures (excellent barrier and recyclability but 100% fossil-based), the hybrid offers a balance of renewable content, plastic reduction, and adequate barrier for dry products.

From a polymer science perspective, this study contributes to understanding the humidity-dependent permeability of EVOH in multilayer configurations where the barrier layer is asymmetrically protected. The observation that OTR increases from 0.56 to 15.2 cm^3^/m^2^/day between 0% and 75% RH is consistent with free-volume theory, where water molecules (kinetic diameter ~0.26 nm) plasticize the EVOH matrix, increasing segmental mobility and reducing tortuosity. The activation energy for oxygen permeation (calculated from Arrhenius plots, Ea = 35 kJ/mol at 0% RH) decreased to 18 kJ/mol upon humidification (75% RH), confirming the plasticization effect. This represents a polymer physics contribution beyond simple packaging performance reporting.

While this study did not include experimental testing of monomaterial PE/EVOH/PE structures, literature values (Table 10) indicate comparable OTR performance (0.5–2.0 cm^3^/m^2^/day) and superior recyclability. The hybrid’s advantage lies in its 37% reduction in plastic content and its paper-based, renewable content, positioning it as a transitional solution when fossil-based monomaterials are undesirable.

### 4.8. Limitations and Generalizability

Although this study demonstrates the feasibility of dry herbal infusions, further validation is necessary to assess generalizability across additional product categories. High-fat products may interact differently with the paper substrate, while acidic or alkaline foods could compromise adhesive integrity. Frozen applications require performance assessment at low temperatures. Furthermore, the focus on laboratory-scale production limits insight into real-world variability and processing challenges, which pilot-scale trials are better positioned to address.

These findings are most applicable to markets with established paper recycling infrastructure and to consumers who prioritize sustainability and are willing to accept moderate cost premiums. In regions lacking such infrastructure, the environmental benefits depend on the degradation pathway, leading to distinct trade-offs in methane emissions.

The life cycle assessment (LCA) in this study utilized regionally representative end-of-life (EoL) scenarios. Conducting a comprehensive comparative LCA with identically modeled EoL pathways, although beyond the current scope due to software access limitations, constitutes a promising direction for future research. Sensitivity analysis demonstrates that the primary conclusions regarding material production advantages are robust across varying EoL assumptions. Future studies should also include dynamic modeling of degradation products and their fate across diverse waste management systems, as well as pilot-scale production trials to assess processability and economic feasibility under industrial conditions.

Future studies should also include thermal characterization (DSC, TGA) and melt flow index measurements of the separated polymer fractions after repulping to assess their suitability for reprocessing into secondary plastic products.

## 5. Conclusions

A circular economy can be achieved through the systematic redesign of traditional laminated flexible packaging into hybrid paper-and-polymer alternatives (hybrid design). Utilizing cellulose as the base substrate in conjunction with high-barrier EVOH-LDPE co-extrusion has resulted in a 37% reduction in virgin plastic and a 50% reduction in adhesive and solvent usage, with an overall 47% reduction in environmental impact in producing these materials, regardless of their end-of-life scenarios.

The hybrid design also provided the functional performance required for dry-food applications, meeting all ASTM destructive distribution test requirements (drop, vacuum, and pressure). In addition, it achieved a superior oxygen barrier compared to trilaminated flexible packaging (0.56 cm^3^/m^2^/day at 0% RH). While this hybrid design has less ductility than fossil-based materials, it is sufficiently robust mechanically to be produced in a stand-up pouch format when packaged with nonfragile products.

To ensure large-scale industrialization of the hybrid design materials, additional areas of future research may include:

- Additional Dynamic Durability Testing: Evaluating the potential for flex-crack propagation during repeated high-velocity handling by conducting cyclic flexural testing (e.g., Gelbo-flex per ASTM F392) under simulated commercial use conditions.
- Optimization of Processing Equipment: Evaluating and optimizing the processing and packaging equipment at both industrial line speed and heat-sealing window to optimize packaging output.
- Exploration of alternative biopolymer substrates (PLA nonwovens, PHA fiber mats, bacterial cellulose) to improve ductility while maintaining biodegradability.
- Development of fully bio-based barrier layers to replace EVOH, potentially using chitosan or modified starch nanocomposites.

## Figures and Tables

**Table 1:** Comparison of conventional trilaminate, hybrid paper-based laminate, and commercial high-barrier flexible packaging structures for oxygen-sensitive dry foods.

Structure	OTR (cm^3^/m^2^/Day, 0% RH)	WVTR (g/m^2^/Day, 38 °C/90% RH)	Recyclability Classification (CEFLEX)	Relative Cost Index (Conventional = 1.0)	Key Sustainability Notes
PET/PET/LDPE (Baseline)	35.38 ± 2.1	4.85 ± 0.3	Not recyclable (multi-material)	1	Non-recyclable, fossil-based
PET/met-PET/PE	0.10–0.50	0.50–1.20	Not recyclable (metallized layer)	1.20–1.50	High barrier, but non-recyclable
PET/Al/PE	<0.05	<0.10	Not recyclable (aluminum layer)	1.50–2.00	Excellent barrier, but non-recyclable
PE–EVOH–PE (Monomaterial)	0.50–2.00	1.00–1.50	Recyclable (PE stream)	1.10–1.30	Recyclable, but 100% fossil-based
Hybrid Paper-Based (This work)	0.56 ± 0.05	1.22 ± 0.08	Partially recyclable (paper stream)	1.22–1.30	37% plastic reduction, paper substrate

Note: The monomaterial OTR values reported here refer to high-barrier structures containing EVOH. A PE-only monomaterial (without EVOH) would exhibit OTR ≈ 35 cm^3^/m^2^/day, similar to the conventional baseline. Cost index based on 2024 market data in Mexico. Values represent mean ± standard deviation (*n* = 5).

**Table 2:** Standard quality parameters for flexible packaging in coil and Doypack^®^ formats.

Quality Requirement	Units	International Standards
Base weight	g/m^2^	--
Thickness	microns	ASTM D6988-21 [25]
Lamination strength	gf	ASTM F88/F88M-23 [26]
Seal strength	gf	ASTM F88/F88M-23 [26]
Tensile strength	gf	ASTM D882-18 [27]
Elongation percentage	mm	ASTM D882-18 [27]
Coefficient of friction	Non dimensional	ASTM D1894-14 [28]
Vacuum tightness	Non dimensional	ASTM D3078-02 [29]
Drop packing resistance	Non dimensional	Based on ISTA 3A [30]
Air pressure packing resistance	Non dimensional	--
Shelf life	Months	--

**Table 3:** Comparative material properties: Conventional trilaminate versus proposed hybrid flexible packaging (stand-up pouch format).

**Trilaminate Structure**	**Thickness [Microns]**	**Base Weight [g/m^2^]**	**Variation %**
Natural polyester	12	16.8	10%
Ink	3	3	5%
Adhesive	3	3	5%
Natural polyester	12	16.8	10%
Adhesive	3	3	5%
LDPE	62.5	60	10%
Total	95.5	102.6	10%
**Hybrid structure** **(Paper and biodegradable plastic)**	**Thickness [microns]**	**Base weight [g/m^2^]**	**Variation %**
Cellulose Paper	50	40	10%
Water-based ink	3	3	5%
Adhesive	3	3	5%
Polyethylene + EVOH + ECO-ONE^®^	62.5	72	10%
Total	118.5	118	10%

Note: Variation % represents the typical manufacturing tolerance for each layer thickness and base weight, based on historical quality-control data from industrial production trials (*n* = 30 batches). These values reflect normal process variability in commercial converting operations. Trilaminate structure material properties were originally presented in [1].

**Table 4:** Comparative analysis of mechanical and barrier properties between conventional trilaminate and paper-polymer hybrid structures.

Quality Requirement	Current Trilaminate Structure	Proposed Hybrid Structure	Units	*p*-Value
Base weight	102.6 ± 1.2	118.0 ± 1.5	g/m^2^	<0.01
Thickness	95.6 ± 1.51	125.8 ± 1.30	microns	<0.01
Lamination strength	154.2 ± 37.77	212.6 ± 35.78	gf	0.012
Seal strength	3779.4 ± 1678.26	3700.4 ± 284.59	gf	0.45
Tensile strength (MD)	13,162.4 ± 1803.5	6074 ± 1903.2	gf	<0.01
Tensile strength (TD)	12,420.8 ± 1461.34	6229.4 ± 344.33	gf	<0.01
Elongation % (MD)	37.124 ± 6.052	0.628 ± 0.366	%	<0.01
Elongation % (TD)	31.346 ± 12.74	2.564 ± 0.488	%	<0.01
Friction coefficient (ST)	0.19 ± 0.015	0.2166 ± 0.041	dimensionless	0.08
Friction coefficient (DI)	0.13 ± 0.052	0.13 ± 0.026	dimensionless	0.95
Packaging drop test	5/5 passed	5/5 passed	pass/fail	----
Vacuum tightness	3/3 passed	3/3 passed	pass/fail	----
Air pressure test	5/5 passed	5/5 passed	pass/fail	----
Shelf life	Pass	Pass	pass/fail	----

MD: Machine direction, TD: Transverse direction, ST: Static friction coefficient, DI: Dynamic friction coefficient. Sample sizes: *n* = 5 for mechanical tests; *n* = 3 for destructive tests (drop, vacuum, pressure). Barrier tests: *n* = 5.

**Table 5:** Standardized barrier properties of conventional trilaminate and hybrid paper-based Doypack^®^ structures.

Property	Test Standard	Conditions	Conventional Trilaminate	Hybrid Paper-Based	*p*-Value
OTR (cm^3^/m^2^/day)	ASTM D3985	23 °C, 0% RH	35.38 ± 2.1	0.56 ± 0.05	<0.01
WVTR (g/m^2^/day)	ASTM F1249	38 °C, 90% RH	4.85 ± 0.3	1.22 ± 0.08	<0.01

Note: Values represent mean ± SD (*n* = 5). Statistical significance determined via Student’s *t*-test.

**Table 6:** Estimated Environmental Impact Reduction Across Different EoL Scenarios.

Impact Category	Baseline (Mixed) Scenario Reduction	Scenario A (Both Landfills) Reduction	Scenario B (Both Incineration) Reduction	Primary Driver
Overall Impact (Single Score)	47.3%	53.1%	43.8%	Material Production
Global Warming Potential (GWP100)	42%	48.5%	40.2%	Plastic & Solvent Reduction
Fossil Resource Scarcity	51%	54.3%	49.7%	Plastic Reduction
Freshwater Eutrophication	38%	41.2%	36.5%	Solvent Elimination

Note: Values for Scenarios A and B represent directional estimates based on contribution analysis rather than fully recalculated LCA results, due to software constraints. These estimates indicate the sensitivity of results to EoL assumptions but should not be interpreted as precise LCA outcomes. The baseline scenario (mixed) represents the fully modeled results with 95% confidence intervals from Monte Carlo simulation (±5.2% for GWP, ±6.8% for single score).

**Table 7:** Humidity-dependent oxygen transmission rate (OTR) for conventional trilaminate and hybrid paper-based structures.

Relative Humidity (%)	OTR Hybrid (cm^3^/m^2^/Day)	OTR Conventional (cm^3^/m^2^/Day)
0%	0.56 ± 0.05	35.38 ± 2.10
50%	2.31 ± 0.41	36.12 ± 2.33
75%	15.21 ± 2.14	36.84 ± 2.42

Note: Values represent mean ± SD (*n* = 3). RH = relative humidity.

**Table 8:** Water vapor transmission rate (WVTR) for conventional trilaminate and hybrid paper-based structures at varying relative humidity.

Relative Humidity (%)	OTR Hybrid (cm^3^/m^2^/Day)	OTR Conventional (cm^3^/m^2^/Day)
50%	1.18 ± 0.09	4.72 ± 0.31
75%	1.25 ± 0.11	4.91 ± 0.35
90%	1.22 ± 0.08	4.85 ± 0.30

Note: Values represent mean ± SD (*n* = 3). Testing conducted at 38 °C per ASTM F1249.

**Table 9:** Supplementary mechanical properties for puncture, tear, and impact resistance of conventional trilaminate and hybrid paper-based structures.

Test	Standard	Sample Size (*n*)	Pass/Fail Threshold	Conventional Trilaminate	Hybrid Paper-Based
Puncture Resistance (N)	ASTM D5748	5	>30 N	45.2 ± 3.1	38.7 ± 2.8
Tear Strength (mN)	ASTM D1922	5	>250 mN	420 ± 25	310 ± 30
Dart Impact (g)	ASTM D1709	5	>80 g	125 ± 8	95 ± 10

Note: All tests conducted at 23 °C and 50% RH. Pass thresholds in accordance with industry guidelines for flexible food packaging. Both structures met all minimum requirements.

**Table 10:** Comparative positioning of the hybrid paper-polymer laminate versus alternative sustainable packaging strategies.

Parameter	Hybrid Paper-Polymer (This Study)	Monomaterial PE/EVOH	Paper + Dispersion Coating	Compostable Biopolymers (e.g., PLA/PBAT)
Renewable Content	High (paper substrate)	None (fossil-based)	High (paper substrate)	Variable (bio-based feedstocks)
Plastic Reduction vs. Conventional	37%	0–20% (down-gauging)	50–70%	100% (if fully bio-based)
O_2_ Barrier (cm^3^/m^2^/day)	0.56 (excellent)	0.5–2.0 (excellent)	10–50 (moderate to poor)	50–200 (poor)
Moisture Barrier (WVTR g/m^2^/day)	1.22 (good)	1.0–1.5 (good)	5–15 (moderate)	10–20 (poor)
Recyclability	Partial (paper stream, 78.5% yield)	High (PE stream)	High (paper stream)	None (compost only)
Compostability	No (anaerobic degradation only)	No	No (coatings hinder)	Yes (industrial)
Key Advantage	Balanced plastic reduction + barrier	Recycling compatibility	Paper recyclability + simplicity	Clear end-of-life (compost)
Key Limitation	Complex recycling; infrastructure-dependent	Fossil-based; limited source reduction	Poor oxygen barrier	Poor moisture barrier; cost

## Data Availability

The original contributions presented in this study are included in the article. Further inquiries can be directed to the corresponding authors.
